# Supplementary material for: Xenosiderophore transporter gene expression and clade-specific filamentation in Candida auris killifish (Aphanius dispar) infection
Source: Commun Biol. 2025 Dec 19;8:1790. doi: 10.1038/s42003-025-09321-z (PMC12717273; doi:10.1038/s42003-025-09321-z)
Supplement: Supplementary file 2 — Supplementary Information [file 42003_2025_9321_MOESM2_ESM.pdf]

Supplementary Information Appendix

Supplementary Note 1

To examine orthologues across reference genomes for each clade (**Figure S4**), we identified 5,324 orthogroups across clades I-V with outliers *C. haemulonii* and *C. albicans* containing 95.4% of all genes (*n* = 38,345 genes, **Figure S4A-B**). The use of clade-specific reference genomes identified higher numbers of DEGs (692 *vs* 499 up-regulated and 714 *vs* 523 down-regulated genes) including genes within the accessory genome (55 *vs* 40 up-regulated and 57 *vs* 45 down-regulated, **Figure S4C**), which we defined as orthogroups of genes not found in every clade. The core genome across all three species and five clades of *C. auris* included 3619 single copy orthologues ($\bar{x}$ = 66.9% *C. auris* genome per clade) and a further 869 single copy orthologues in *C. auris* ($\bar{x}$ = 82.9% *C. auris* genome per clade), of which 133 were unique to *C. auris*. The remaining core genome included 486-585 multiple copy orthologues shared across all species and 110-121 in *C. auris*, of which 17-19 were present in each clade ($\bar{x}$ = 643.2 genes per clade), bringing the total estimated core genome size to 5131.2/5413.2 genes per clade ($\bar{x}$ = 94.8%). A single copy orthologue tree confirmed the basal status of clade V, and synteny plotting was consistent with limited transversions/inversions within *C. auris* outwith clade II (**Figure S4A**). These findings are consistent with a highly conserved core yeast genome across *Candida* species, especially within the *Metschinikowiaceae* clade, which appears to contain a degree of preserved genome structure.

We observed high correlation between log-fold change values obtained from clade-specific and core (B8441) reference genomes (Pearson's ≥0.95, p <0.001 per comparison). Among DEGs that had no orthologues in the clade I reference genome (**Figure S4D**), the highest number of clade-unique genes that were differentially expressed was identified in clade IV; other DEGs included genes involved in cell wall, intracellular transport, metabolism, transcription/translation and transmembrane transport (**Data S3**). In terms of siderophore transporters, we additionally identified a clade IV unique gene associated with a siderophore transport GO term that was differentially expressed at both time-points of infection (CJJ09_005327). Overall, the *in vivo* expression profile across five *C. auris* clades indicated a potential role for the expanded gene family of siderophore transporters during *C. auris* in-host survival and pathogenicity, which was up-regulated compared to growth in nutrient-rich laboratory media.

Comparing transcript levels across *MTL* genes in all five clades, we observed poorer detection of ***MTLα*** transcripts when aligned to the clade I reference genome, as expected (**Figure S6A**). Direct transcript-level testing, with correction for multiple testing to minimise false positives, indicated that only up-regulation of *PIKA* and *PGA30* (48 HPI) and down-regulation of *THI4* (24 HPI)

and *MDR1* (at both time-points) were significantly different across the set of 10 DEGs shared across all comparisons (**Figure S6B**).

We used the clade I reference genome to annotate functional domains and discover enriched gene families, revealing enrichment of up-regulated hypha-regulated cell wall GPI-anchored proteins in more virulent clades (**Figure 6**), driven by three pairs of orthologues of *IFF4*, *HYR3* and *RBR3* (**Data S3**). Four secretory lipase *LIP1* paralogues drove an enrichment of the related PFAM domain for clade I *vs* III at 24 HPI. When comparing clade I and IV infection to clade II and 48 HPI, we also observed up-regulated nutrient transporters including a 7 transmembrane domain putative ferric reductase/iron importer, *CFL4*, as well as ferric reductase, *FRE3*, and siderophore transporter orthologues *SIT1_1948*, *SIT1_2241* and *SIT1_4097*. Cell wall components, non-mating *MTL* locus genes such as *PIKA*, and genes involved in iron and siderophore transport are therefore potential modulators of strain-specific differences in virulence.

Supplementary Figures

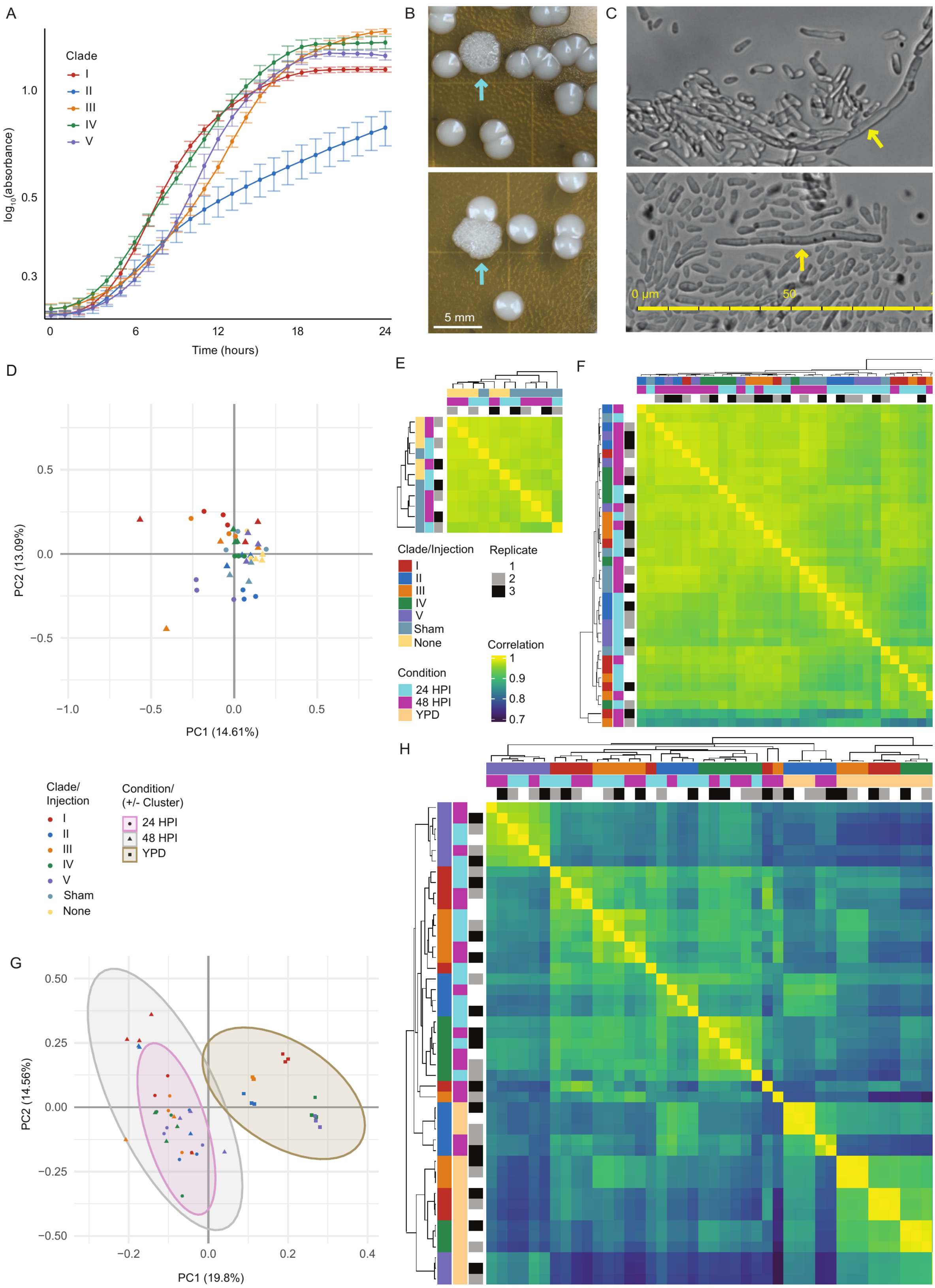

Figure S1: Profiles of *C. auris* growth, morphotype, morphology, and gene expression. **(A)** Growth curves for five representative strains of *C. auris* over 24 h *in vitro*. **(B)** Rough colonies demonstrated on YPD-agar after recovery from AK infection in clade V only (blue arrows). **(C)**

Filamentous forms (yellow arrows) isolated from rough colonies and imaged on light microscopy in distilled de-ionised water. **(D)** Principle components analysis (PCA) of fragments per kilobase per million (FPKM) gene expression for AK host transcription. **(E)** Correlation matrix for individual replicates' log_2-transformed transcript counts per million for AK responses to sham injection and no injection **(F)** Correlation matrix as above for AK responses to *C. auris* infection and sham injection. **(G)** PCA of FPKM gene expression for *C. auris* pathogen transcription. **(H)** Correlation matrix as above for *C. auris* gene expression *in vivo* and *in vivo* across five clades.

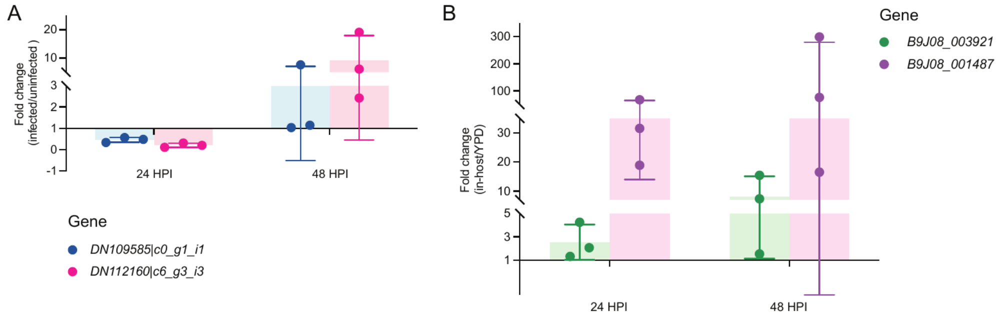

Figure S2: Comparing fold-change by qPCR for RNA recovered from clade IV infected embryos to confirm host and pathogen findings. **(A)** Fold change for two *HMOX* genes, DN109585|c0_g1_i1 and DN112160|c6_g3_i3. Bars represent mean of three replicates; error bars represent standard deviation. **(B)** Fold change for two *XTC* genes. Bars represent mean of three replicates; error bars represent standard deviation.

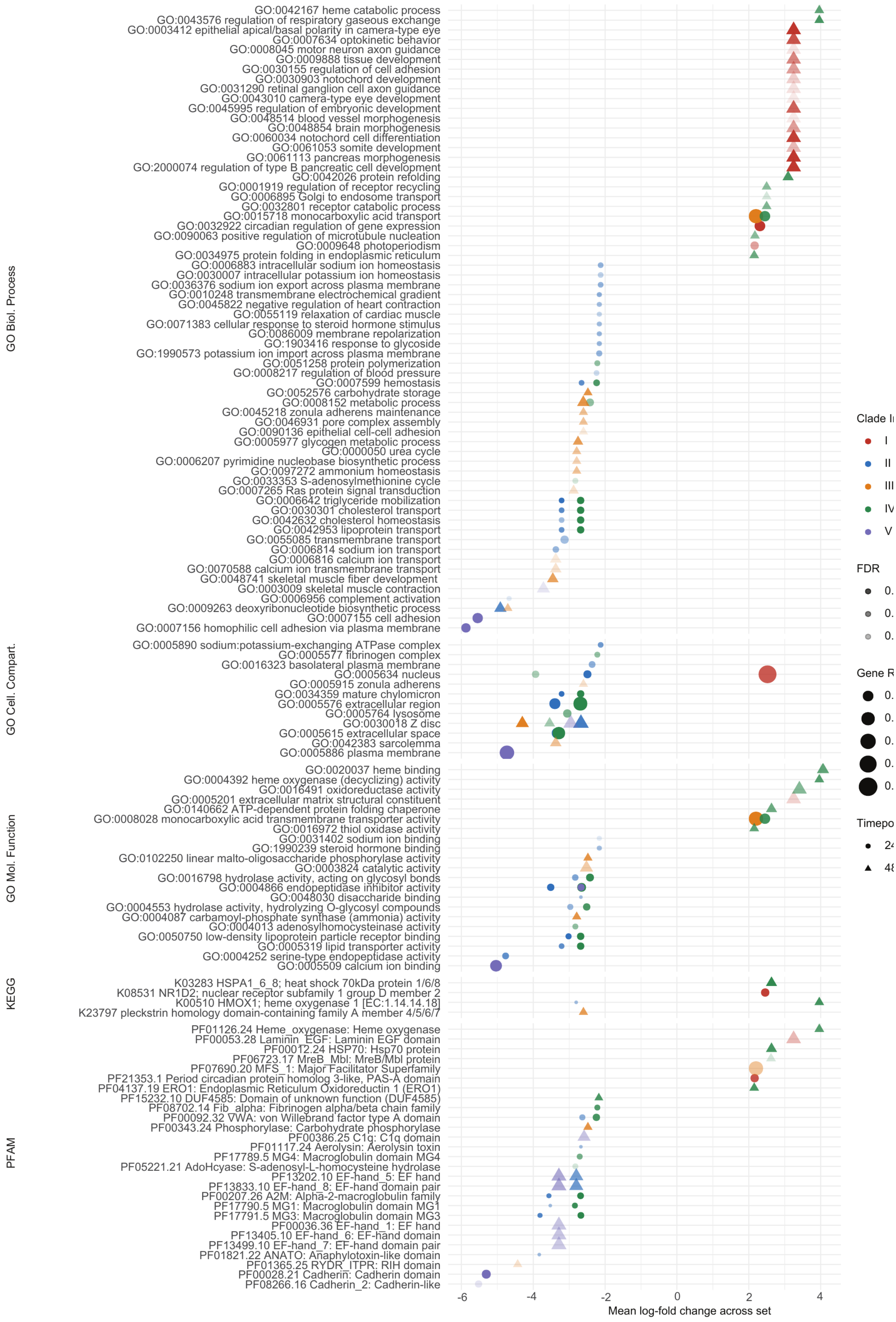
GO Biol. Process
GO:0042167 heme catabolic process
GO:0043576 regulation of respiratory gaseous exchange
GO:0003412 epithelial apical/basal polarity in camera-type eye
GO:0007634 optokinetic behavior
GO:0008045 motor neuron axon guidance
GO:0009888 tissue development
GO:0030155 regulation of cell adhesion
GO:0030903 notochord development
GO:0031290 retinal ganglion cell axon guidance
GO:0043010 camera-type eye development
GO:0045995 regulation of embryonic development
GO:0048514 blood vessel morphogenesis
GO:0048854 brain morphogenesis
GO:0060034 notochord cell differentiation
GO:0061053 somite development
GO:0061113 pancreas morphogenesis
GO:2000074 regulation of type B pancreatic cell development
GO:0042026 protein refolding
GO:0001919 regulation of receptor recycling
GO:0006895 Golgi to endosome transport
GO:0032801 receptor catabolic process
GO:0015718 monocarboxylic acid transport
GO:0032922 circadian regulation of gene expression
GO:0090063 positive regulation of microtubule nucleation
GO:0009648 photoperiodism
GO:0034975 protein folding in endoplasmic reticulum
GO:0006883 intracellular sodium ion homeostasis
GO:0030007 intracellular potassium ion homeostasis
GO:0036376 sodium ion export across plasma membrane
GO:0010248 transmembrane electrochemical gradient
GO:0045822 negative regulation of heart contraction
GO:0055119 relaxation of cardiac muscle
GO:0071383 cellular response to steroid hormone stimulus
GO:0086009 membrane repolarization
GO:1903416 response to glycoside
GO:1990573 potassium ion import across plasma membrane
GO:0051258 protein polymerization
GO:0008217 regulation of blood pressure
GO:0007599 hemostasis
GO:0052576 carbohydrate storage
GO:0008152 metabolic process
GO:0045218 zonula adherens maintenance
GO:0046931 pore complex assembly
GO:0090136 epithelial cell-cell adhesion
GO:0005977 glycogen metabolic process
GO:0000050 urea cycle
GO:0006207 pyrimidine nucleobase biosynthetic process
GO:0097272 ammonium homeostasis
GO:0033353 S-adenosylmethionine cycle
GO:0007265 Ras protein signal transduction
GO:0006642 triglyceride mobilization
GO:0030301 cholesterol transport
GO:0042632 cholesterol homeostasis
GO:0042953 lipoprotein transport
GO:0055085 transmembrane transport
GO:0006814 sodium ion transport
GO:0006816 calcium ion transport
GO:0070588 calcium ion transmembrane transport
GO:0048741 skeletal muscle fiber development
GO:0003009 skeletal muscle contraction
GO:0006956 complement activation
GO:0009263 deoxyribonucleotide biosynthetic process
GO:0007155 cell adhesion
GO:0007156 homophilic cell adhesion via plasma membrane
GO Cell. Compart.
GO:0005890 sodium:potassium-exchanging ATPase complex
GO:0005577 fibrinogen complex
GO:0016323 basolateral plasma membrane
GO:0005634 nucleus
GO:0005915 zonula adherens
GO:0034359 mature chylomicron
GO:0005576 extracellular region
GO:0005764 lysosome
GO:0030018 Z disc
GO:0005615 extracellular space
GO:0042383 sarcolemma
GO:0005886 plasma membrane
GO Mol. Function
GO:0020037 heme binding
GO:0004392 heme oxygenase (decyclizing) activity
GO:0016491 oxidoreductase activity
GO:0005201 extracellular matrix structural constituent
GO:0140662 ATP-dependent protein folding chaperone
GO:0008028 monocarboxylic acid transmembrane transporter activity
GO:0016972 thiol oxidase activity
GO:0031402 sodium ion binding
GO:1990239 steroid hormone binding
GO:0102250 linear malto-oligosaccharide phosphorylase activity
GO:0003824 catalytic activity
GO:0016798 hydrolase activity, acting on glycosyl bonds
GO:0004866 endopeptidase inhibitor activity
GO:0048030 disaccharide binding
GO:0004553 hydrolase activity, hydrolyzing O-glycosyl compounds
GO:0004087 carbamoyl-phosphate synthase (ammonia) activity
GO:0004013 adenosylhomocysteinase activity
GO:0050750 low-density lipoprotein particle receptor binding
GO:0005319 lipid transporter activity
GO:0004252 serine-type endopeptidase activity
GO:0005509 calcium ion binding
KEGG
K03283 HSPA1_6_8; heat shock 70kDa protein 1/6/8
K08531 NR1D2; nuclear receptor subfamily 1 group D member 2
K00510 HMOX1; heme oxygenase 1 [EC:1.14.14.18]
K23797 pleckstrin homology domain-containing family A member 4/5/6/7
PFAM
PF01126.24 Heme_oxygenase: Heme oxygenase
PF00053.28 Laminin_EGF: Laminin EGF domain
PF00012.24 HSP70: Hsp70 protein
PF06723.17 MreB_Mbl: MreB/Mbl protein
PF07690.20 MFS_1: Major Facilitator Superfamily
PF21353.1 Period circadian protein homolog 3-like, PAS-A domain
PF04137.19 ERO1: Endoplasmic Reticulum Oxidoreductin 1 (ERO1)
PF15232.10 DUF4585: Domain of unknown function (DUF4585)
PF08702.14 Fib_alpha: Fibrinogen alpha/beta chain family
PF00092.32 VWA: von Willebrand factor type A domain
PF00343.24 Phosphorylase: Carbohydrate phosphorylase
PF00386.25 C1q: C1q domain
PF01117.24 Aerolysin: Aerolysin toxin
PF17789.5 MG4: Macroglobulin domain MG4
PF05221.21 AdoHcyase: S-adenosyl-L-homocysteine hydrolase
PF13202.10 EF-hand_5: EF hand
PF13833.10 EF-hand_8: EF-hand domain pair
PF00207.26 A2M: Alpha-2-macroglobulin family
PF17790.5 MG1: Macroglobulin domain MG1
PF17791.5 MG3: Macroglobulin domain MG3
PF00036.36 EF-hand_1: EF hand
PF13405.10 EF-hand_6: EF-hand domain
PF13499.10 EF-hand_7: EF-hand domain pair
PF01821.22 ANATO: Anaphylotoxin-like domain
PF01365.25 RYDR_ITPR: RIH domain
PF00028.21 Cadherin: Cadherin domain
PF08266.16 Cadherin_2: Cadherin-like
-6
-4
-2
0
2
4
Mean log-fold change across set
Clade I
I
II
III
IV
V
FDR
0.
0.
0.
Gene R
0.
0.
0.
0.
0.
Timepo
24
48

Figure S3: *A. dispar* gene ontology, pathway and domain enrichment during infection *vs* sham control: The GJEY01 *A. dispar* transcripts were annotated for Gene Ontology (GO) terms, Kyoto Encyclopaedia of Genes and Genome (KEGG) pathways and PFAM domains. Enrichment testing was performed with Fisher's exact test with Benjamini-Hochberg (BH) correction for multiple testing with a false discovery rate (FDR) cut-off of 0.001. Mean log-fold change and ratio of genes in that set was calculated for genes in each set possessing each feature.

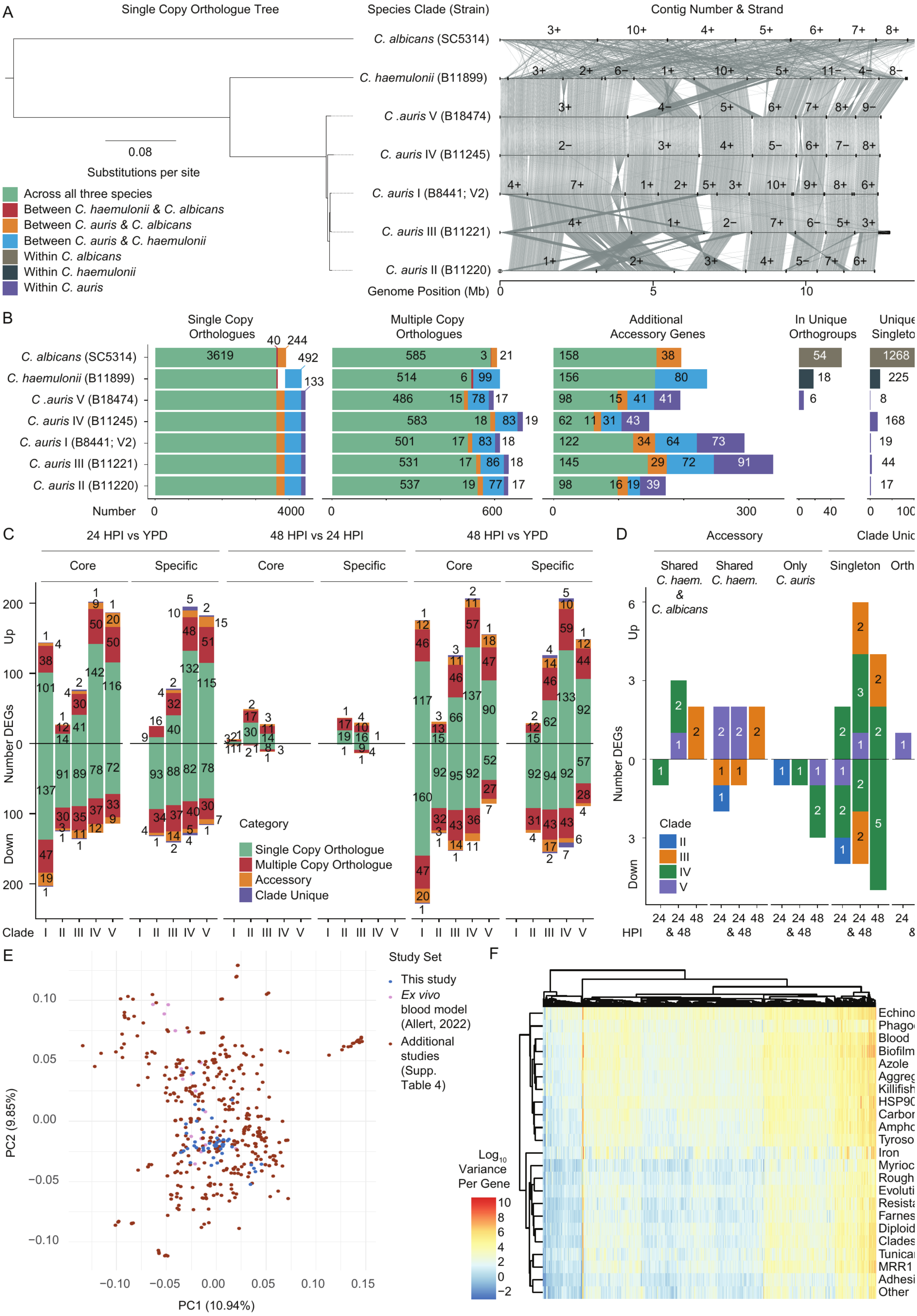

Figure S4: Contextualising differential gene expression across orthogroups and broader RNA-seq datasets. **(A)** Single copy orthologue phylogeny synteny. **(B)** Shared/unique orthologue counts. **(C)**

Differential gene expression for *in vivo vs in vitro* comparisons, comparing numbers of DEGs identified when using a single core reference genome (B8441, clade I) or a clade-specific reference genome for each individual clade. **(D)** Differential expression of specific genes not present in core reference genome. **(E)** Per-locus variance of single copy orthologues from RNA-seq meta-analysis of 36 public datasets for *C. auris* transcriptome expression. **(F)** Hierarchical clustering of per-gene variance, calculated for all B8441 core genes for each study.

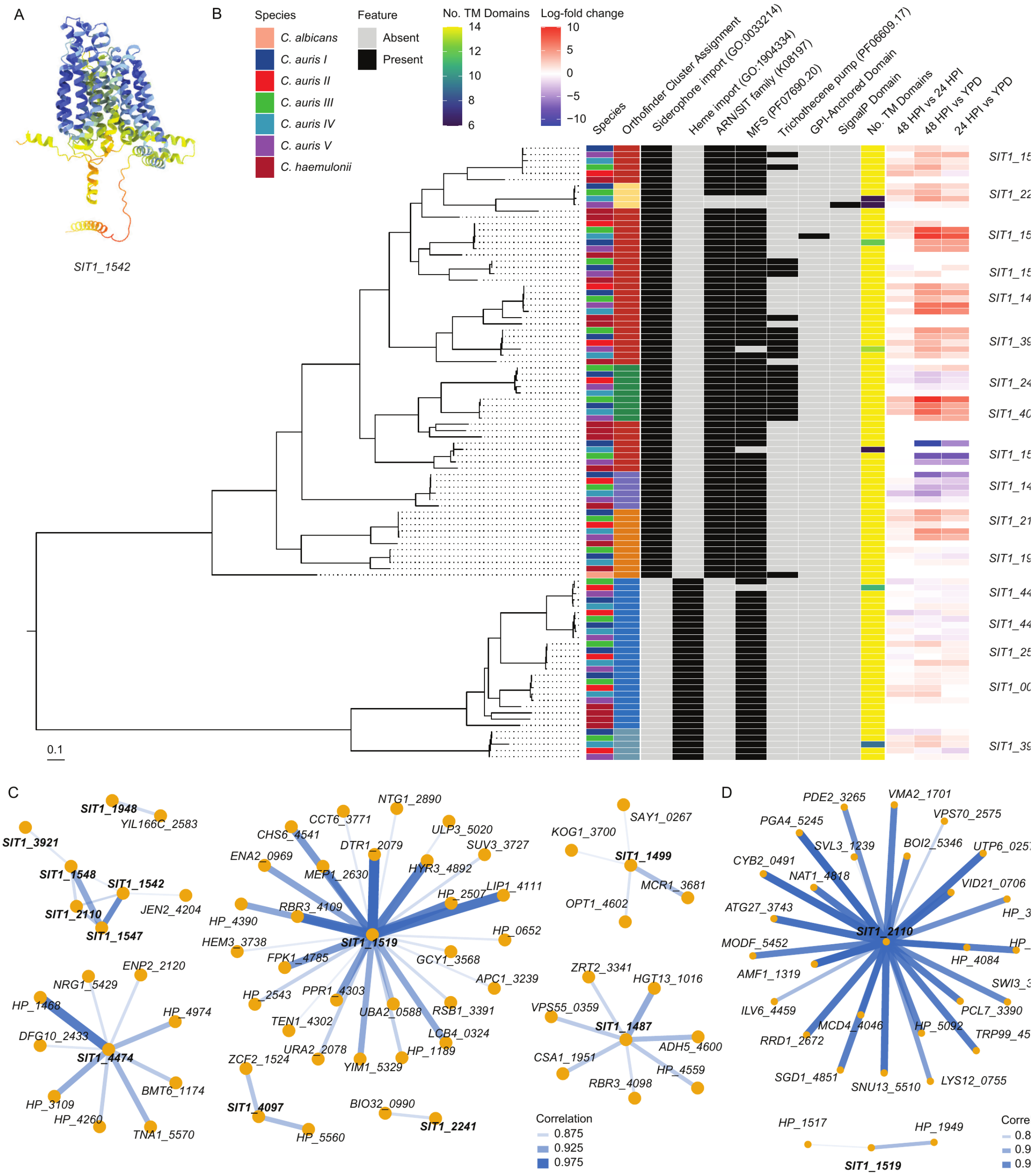

Figure S5: Xenosiderophore Transporter Analysis: **(A)** AlphaFold 3 predicted protein folding for peptide sequences for *SIT1* 1542, illustrating the conserved 14-transmembrane domain similarity across the families. **(B)** RAXML phylogeny using 1000 bootstraps and LG4X model of Muscle-aligned protein sequences for orthologues of *C. albicans* Siderophore Transport 1 *SIT1* gene. Scale bar indicates number of substitutions per site. Orthofinder cluster assignment notes which grouping each gene was assigned to by our Synima/Orthofinder pipeline. **(C)** Pearson's correlation co-expression of associated gene expression involving siderophore transport candidate genes. **(D)** Pearson's correlation co-expression across available RNA-seq data from meta-analysis of 35 studies (**Data S4**), demonstrating a different set of transcriptional co-expression indicated by external experimental data.

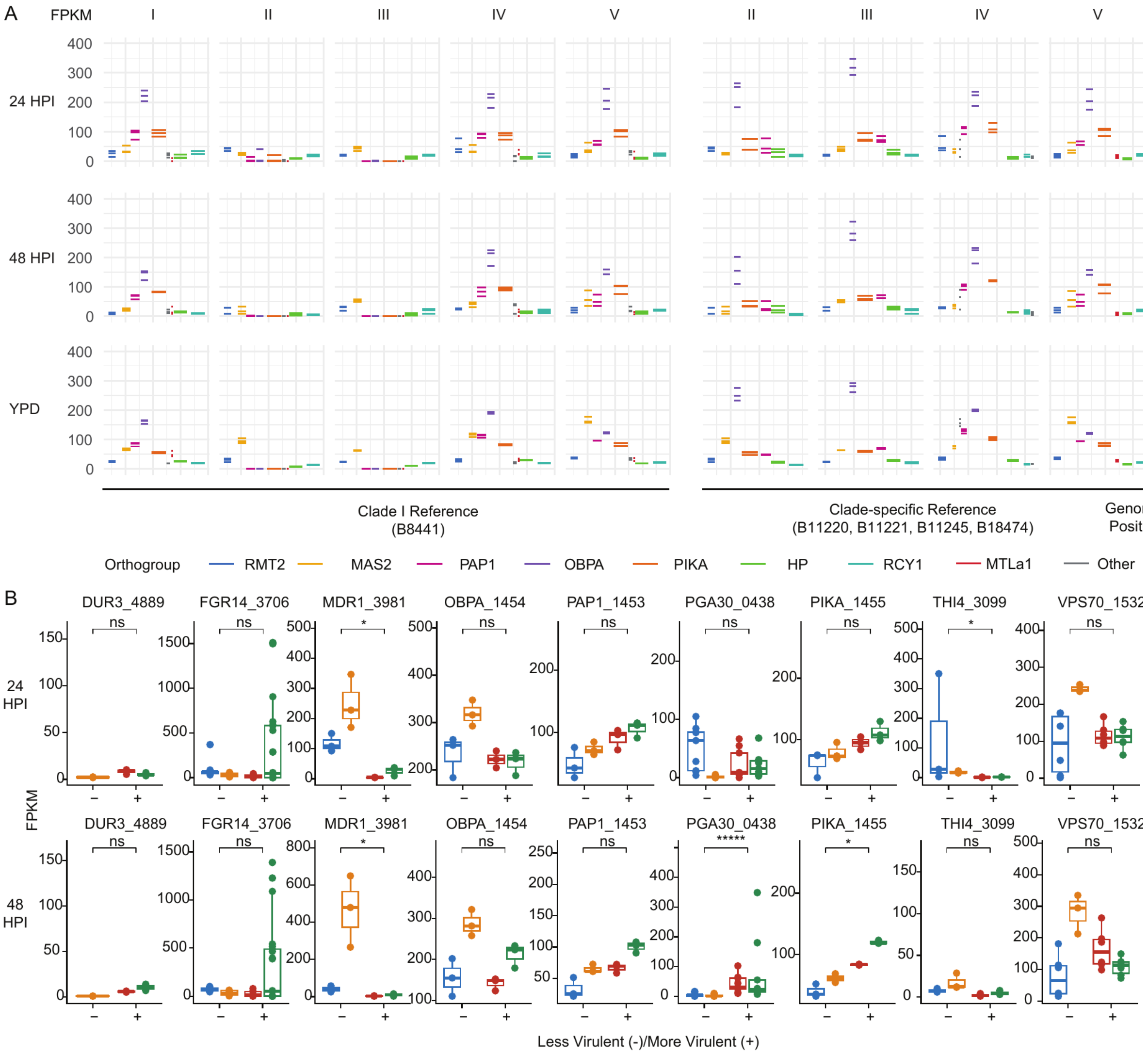

Figure S6: *C. auris* Mating-Type Locus Gene Expression: **(A)** Transcripts (fragments per kilobase per million, FPKM) for orthogroups aligned to the same reference genome (left-hand panels) and clade-specific reference genomes (right-hand panels), indicating similar transcript levels between

clades for e.g. *PIKA*, *OBPA* and *PAP1* non-mating genes when aligned to clade-specific reference genomes. **(B)** Comparing individual transcript levels for each orthogroup in more *vs* less virulent clades *via* Wilcoxon signed-rank test with Bonferroni correction to minimise false positives, demonstrating that only down-regulation of *THI4* (at 24 HPI), *MRD1* (at both time-points), and up-regulation of *PGA30* and *PIKA* were significantly different. Significance levels are as follows: * p ≤0.05, ** ≤0.01, *** ≤0.001, **** ≤0.0001, ***** ≤0.00001.

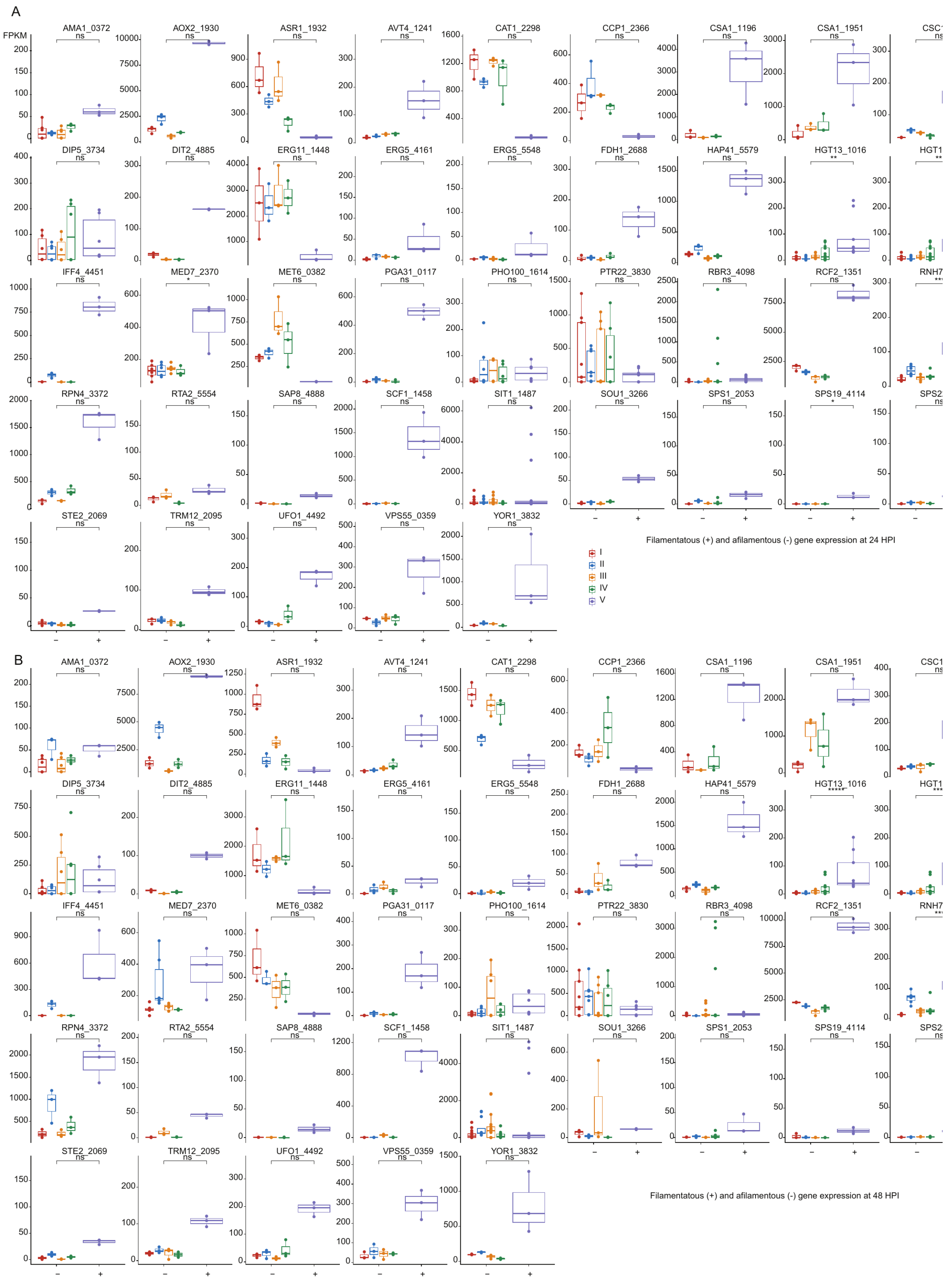

Figure S7: Comparing individual transcript levels for each orthogroup in filamentous clade V *vs* all other clades *via* Wilcoxon signed-rank test with Bonferroni correction to minimise false positives, demonstrating at 24 HPI **(A)** and 48 HPI **(B)**, excluding any genes that were assigned to be

‘hypothetical proteins’ only, up-regulation of two *HGT13* orthologues and *RNH70* were significantly different at both time-points, with up-regulation of *MED7* and *SPS19* at 24 HPI. Significance levels are as follows: * p ≤0.05, ** ≤0.01, *** ≤0.001, **** ≤0.0001, ***** ≤0.00001.

Supplementary Tables

Data S1. Clade-representative *Candida auris* strains used in this study: Representative strain for each major clade of *C. auris* used in the study, with notes on human disease source, country of discovery, and date of isolation. Mortality statistics are given in terms of absolute numbers of embryos over biological triplicate experiments. Additional significance testing is also noted for pairwise comparisons by Log-Rank test with Benjamini-Hochberg (BH) correction.

Data S2: Differentially expressed genes in *A. dispar* microinjection: All differentially expressed genes (DEGs) for *A. dispar* for (upper rows) sham injection *vs* no injection and (lower rows) *C. auris* injection *vs* sham injection. Log-fold change and -$\log_{10}$(false discovery rate) are calculated across all clades as a mean value for *C. auris* injection. Significant DEGs for each set of embryos injected with each clade are given (1 = significant, 0 = not significant), and the total number of clades where DEGs where significant is also given.

Data S3. Differentially expressed genes in *C. auris* infection: **(A)** Combined set of differentially expressed genes (DEGs) expressed by five clades during infection. Each set of DEGs expressed *in vivo vs in vitro* for five clades was combined, resulting in a set of genes that were specific to *C. auris* infection at these timepoints. **(B)** Combined set of DEGs between more virulent and less virulent clades: Each set of DEGs between clades I & IV (more virulent) and II & III (less virulent) was compared at 24 HPI and 48 HPI, resulting in a combined set expressed in infection. **(C)** Combined DEG Sets: between filamentous clade V and all other clades: Each set of DEGs between clade V and all each clades I-IV was compared at 24 HPI and 48 HPI, resulting in a combined set expressed in infection, excluding those significant during growth in YPD broth where clade V was not observed to filament. **(D)** Accessory genes not found in clade I reference genome: DEGs without orthologues in the clade I reference were identified and annotated. **(E)** Log-fold change and FDR across all pathogen gene expression. **(F)** Fragments per kilobase per million across all pathogen gene expression.

Data S4. Publicly available RNA-seq Data on 16th April 2024: A systematic re-analysis of RNA expression across NCBI-available datasets for *C. auris* gene expression, categorised by research theme for analysis (**Figure S4E-F**).
