# Supplementary material for: Xenosiderophore transporter gene expression and clade-specific filamentation in Candida auris killifish (Aphanius dispar) infection
Source: Commun Biol. 2025 Dec 19;8:1790. doi: 10.1038/s42003-025-09321-z (PMC12717273; doi:10.1038/s42003-025-09321-z)
Supplement: Supplementary file 3 — Description of Additional Supplementary Files [file 42003_2025_9321_MOESM3_ESM.pdf]

Description of Additional Supplementary files

File name: Supplementary Data 1

Description: Clade-Representative Candida auris Strains Used In This Study

File name: Supplementary Data 2

Description: Differentially Expressed Genes in A. dispar Microinjection

File name: Supplementary Data 3

Description: Candida auris in host gene expression

File name: Supplementary Data 4

Description: Publicly available transcriptome datasets for Candida auris
