# Supplementary material for: Xenosiderophore transporter gene expression and clade-specific filamentation in Candida auris killifish (Aphanius dispar) infection
Source: Commun Biol. 2025 Dec 19;8:1790. doi: 10.1038/s42003-025-09321-z (PMC12717273; doi:10.1038/s42003-025-09321-z)
Supplement: Supplementary file 5 — Reporting summary [file 42003_2025_9321_MOESM5_ESM.pdf]

nature portfolio

Corresponding author(s): Rhys Farrer. Document checked by Hugh Gifford.

Last updated by author(s): 03/11/2025

Reporting Summary

Nature Portfolio wishes to improve the reproducibility of the work that we publish. This form provides structure for consistency and transparency in reporting. For further information on Nature Portfolio policies, see our Editorial Policies and the Editorial Policy Checklist.

Statistics

For all statistical analyses, confirm that the following items are present in the figure legend, table legend, main text, or Methods section.

n/a	Confirmed	
☐	☒	The exact sample size (*n*) for each experimental group/condition, given as a discrete number and unit of measurement
☐	☒	A statement on whether measurements were taken from distinct samples or whether the same sample was measured repeatedly
☐	☒	The statistical test(s) used AND whether they are one- or two-sided<br>*Only common tests should be described solely by name; describe more complex techniques in the Methods section.*
☒	☐	A description of all covariates tested
☐	☒	A description of any assumptions or corrections, such as tests of normality and adjustment for multiple comparisons
☐	☒	A full description of the statistical parameters including central tendency (e.g. means) or other basic estimates (e.g. regression coefficient) AND variation (e.g. standard deviation) or associated estimates of uncertainty (e.g. confidence intervals)
☐	☒	For null hypothesis testing, the test statistic (e.g. *F*, *t*, *r*) with confidence intervals, effect sizes, degrees of freedom and *P* value noted<br>*Give P values as exact values whenever suitable.*
☒	☐	For Bayesian analysis, information on the choice of priors and Markov chain Monte Carlo settings
☒	☐	For hierarchical and complex designs, identification of the appropriate level for tests and full reporting of outcomes
☒	☐	Estimates of effect sizes (e.g. Cohen's *d*, Pearson's *r*), indicating how they were calculated

Our web collection on statistics for biologists contains articles on many of the points above.

Software and code

Policy information about availability of computer code

Data collection	Data was inputted manually into Microsoft Excel for Mac v16.90 and handled within RStudio 2024.04.2+764.
Data analysis	Extensive package information is available via https://github.com/hughgifford/Arabian_Killifish_C_auris_2024 in the "packages_and_environments" folder, where code used for data analysis and figure generation is publicly available.

For manuscripts utilizing custom algorithms or software that are central to the research but not yet described in published literature, software must be made available to editors and reviewers. We strongly encourage code deposition in a community repository (e.g. GitHub). See the Nature Portfolio guidelines for submitting code & software for further information.

Data

Policy information about availability of data

All manuscripts must include a data availability statement. This statement should provide the following information, where applicable:

- Accession codes, unique identifiers, or web links for publicly available datasets
- A description of any restrictions on data availability
- For clinical datasets or third party data, please ensure that the statement adheres to our policy

Raw reads have been deposited via NCBI/GEO via accession GSE277854. All datasets and code required to make figures are available via Github (https://github.com/hughgifford/Arabian_Killifish_C_auris_2024) as well as via Zenodo (DOI: 10.5281/zenodo.17515669).

Research involving human participants, their data, or biological material

Policy information about studies with human participants or human data. See also policy information about sex, gender (identity/presentation), and sexual orientation and race, ethnicity and racism.

Reporting on sex and gender	NA
Reporting on race, ethnicity, or other socially relevant groupings	NA
Population characteristics	NA
Recruitment	NA
Ethics oversight	NA

Note that full information on the approval of the study protocol must also be provided in the manuscript.

Field-specific reporting

Please select the one below that is the best fit for your research. If you are not sure, read the appropriate sections before making your selection.

☒ Life sciences ☐ Behavioural & social sciences ☐ Ecological, evolutionary & environmental sciences

For a reference copy of the document with all sections, see nature.com/documents/nr-reporting-summary-flat.pdf

Life sciences study design

All studies must disclose on these points even when the disclosure is negative.

Sample size	We used an unblinded prospective virulence study comparing six groups of AK embryo yolk-sac microinjection, including five different clades of C. auris, and one control sham injection as the negative control. We used >= 23 embryos per condition based on our power calculation of σ = 0.25, significance = 0.05, power = 0.9, and δ effect size of 25% mortality (two-sided t-test). After excluding embryos that were unhealthy or did not complete successful microinjection, we included n = 10-13 embryos per group per experiment (total n = 34-37 embryos per group). Each embryo was considered to be an experimental unit (n = 70-72 embryos per experiment, total n = 213 embryos). For estimation of C. auris in-host growth by homogenisation and plating of colony forming units, three embryos per condition per time-point across two (0 and 24 HPI) or three (48 HPI) experiments were used (total n = 126). For time lapse microscopy demonstration of A. dispar yolk-sac collapse and death, representative image sets were chosen from a pilot experiment using a reduced dosage (1/10) of C. auris (n = 4). For histological demonstration of C. auris morphology, one embryo per condition at 24 HPI and 48 HPI each were used in one experiment, and seven embryos per condition plus three embryos without injection in a second experiment (n = 57). RNA was extracted from three embryos per condition per time-point (0, 24 and 48 HPI, n = 54) plus three embryos per time-point without any injection (n = 9, total n = 63) as a control for the impact of microinjection.
Data exclusions	Embryos were inspected for viability in terms of an intact blastocyst without discolouration by stereo dissection microscope (Optech, UK) and incubated at 30 °C (Memmert, Germany) for 72 h with day and night cycles with daily ASW changes and removal of dead, unhealthy or underdeveloped embryos, with a maximum of 30 embryos per standard size 100 mm x 15 mm petri dish (Greiner Bio-One, Austria).<br><br>In order to deliver a dose of approx. 500 yeasts in 10 nL solution, an injection bolus was administered to an estimated diameter of 0.2 – 0.3 mm (10-15% of total embryo diameter) via a single chorion puncture. Any embryos that failed to meet these criteria for injection were excluded.
Replication	RNA was extracted from three embryos per condition per time-point (0, 24 and 48 HPI, n = 54) plus three embryos per time-point without any injection (n = 9, total n = 63) as a control for the impact of microinjection." An additional note not currently contained in the manuscript is that two embryos without any injection were processed in a single sample, resulting in two (rather than three) sets of reads for the "no injection" control for Arabian killifish gene expression.
Randomization	Embryos were randomly chosen from each dish and the sequence of injection for each biological repeat was randomly allocated by computer.
Blinding	There was no blinding in this study, due to the objective end point of cessation of heart beat.

Reporting for specific materials, systems and methods

We require information from authors about some types of materials, experimental systems and methods used in many studies. Here, indicate whether each material, system or method listed is relevant to your study. If you are not sure if a list item applies to your research, read the appropriate section before selecting a response.

Materials & experimental systems

n/a	Involved in the study
☒	☐ Antibodies
☒	☐ Eukaryotic cell lines
☒	☐ Palaeontology and archaeology
☐	☒ Animals and other organisms
☒	☐ Clinical data
☒	☐ Dual use research of concern
☒	☐ Plants

Methods

n/a	Involved in the study
☒	☐ ChIP-seq
☒	☐ Flow cytometry
☒	☐ MRI-based neuroimaging

Animals and other research organisms

Policy information about studies involving animals; ARRIVE guidelines recommended for reporting animal research, and Sex and Gender in Research

Laboratory animals	Aphanius dispar: Wild-type adult AK were kept at the Aquatic Resources Facility, Exeter, in two 28 °C incubation tanks, with 12-hour day and night light cycles and artificial enrichment foliage. Embryos were collected using three plastic netted collection chambers per tank with transfer of all enrichment into tanks, and placed overnight from 16:00 to 09:15 in dark conditions.
Wild animals	No wild animals were used
Reporting on sex	No reporting on sex
Field-collected samples	No field-collected samples
Ethics oversight	Ethical Statement: All animal experiments were performed under Project Licence PP4402521 in compliance with University of Exeter Animal Welfare Ethical Review Board regulations and MRC Center for Medical Mycology Project Review Board, and are reported in line with “Animal Research: Reporting in vivo Experiments” (ARRIVE 2.0) Guidelines.

Note that full information on the approval of the study protocol must also be provided in the manuscript.

Plants

Seed stocks	NA
Novel plant genotypes	NA
Authentication	NA
